# TNF-α in Uveitis: From Bench to Clinic

**DOI:** 10.3389/fphar.2021.740057

**Published:** 2021-11-02

**Authors:** Qi Jiang, Zhaohuai Li, Tianyu Tao, Runping Duan, Xianggui Wang, Wenru Su

**Affiliations:** ^1^ State Key Laboratory of Ophthalmology, Zhongshan Ophthalmic Center, Sun Yat-sen University, Guangzhou, China; ^2^ Eye Center of Xiangya Hospital, Central South University, Changsha, China; ^3^ Hunan Key Laboratory of Ophthalmology, Changsha, China

**Keywords:** uveitis, TNF-α, anti-TNF-α agents, infliximab, adalimumab, golimumab, certolizumab pegol, experimental autoimmune uveitis (EAU)

## Abstract

Uveitis is an inflammation of the iris, ciliary body, vitreous, retina, or choroid, which has been shown to be the first manifestation of numerous systemic diseases. Studies about the immunopathogenesis and treatment of uveitis are helpful to comprehend systemic autoimmune diseases, and delay the progression of systemic autoimmune diseases, respectively. Tumor necrosis factor-alpha (TNF-α), a pleiotropic cytokine, plays a pivotal role in intraocular inflammation based on experimental and clinical data. Evidence of the feasibility of using anti-TNF-α agents for uveitis management has increased. Although there are numerous studies on TNF-α in various autoimmune diseases, the pathological mechanism and research progress of TNF-α in uveitis have not been reviewed. Therefore, the objective of this review is to provide a background on the role of TNF-α in the immunopathogenesis of uveitis, as well as from bench to clinical research progress, to better guide TNF-α-based therapeutics for uveitis.

## Introduction

**FIGURE 1:**
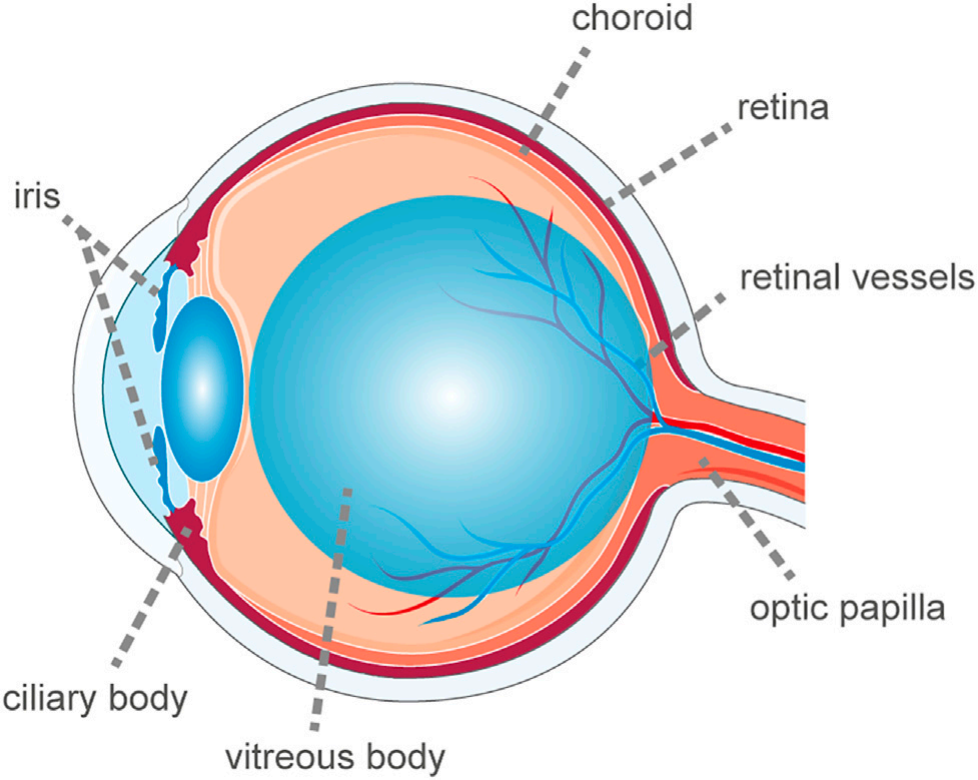
The schematic diagram of ocular anatomy. The term uveitis is broad and encompasses inflammatory damage to the uvea, retina, retinal vessels, vitreous body and optic papilla.

Uveitis is a heterogeneous nosological entity. Although the uvea is defined as the middle membrane (Jabs et al., 2005) of the ocular wall comprising the iris, ciliary body and choroid, the term uveitis is broad and encompasses inflammatory damage to the uvea, retina, retinal vessels, vitreous body, and optic papilla (Figure 1). The incidence of uveitis in the United States is 52.4/100,000 population, with a prevalence of 115/100,000 population (Gritz and Wong, 2004). One of the primary causes of blindness in developing countries is inefficacious control of or untreated uveitis, mainly owing to complications such as macular edema, glaucoma, and retinal ischemia (Dick et al., 2016). Uveitis is often the first manifestation of many systemic autoimmune diseases. According to recent studies, although 23–63% of uveitis cases are idiopathic (Bodaghi et al., 2001; Jakob et al., 2009; Keino et al., 2009; Barisani-Asenbauer et al., 2012; Bajwa et al., 2015; Llorenç et al., 2015; Luca et al., 2018; Hermann et al., 2021; Sonoda et al., 2021), up to 40% of uveitis patients also have systemic autoimmune diseases. The transparency of the eye allows the vascular lesions to be observed directly with the help of certain devices. Direct visualization of the vessels allows ophthalmologists to assess the inflammatory process in depth before serious tissue damage occurs. Therefore, investigating the immunopathological mechanism of uveitis based on a better understanding of autoimmune diseases is important and enables the development of better treatment methods to decrease the blindness rate and control the progression of autoimmune diseases.

Tumor necrosis factor-alpha (TNF-α) is an acidic protein that is mainly produced by macrophages in response to infection and inflammatory irritation. It is related to chronic inflammation and tissue damage in uveitis and is critical for initiating immunity to pathogens (Vassalli, 1992). TNF receptor I (TNFR1) and TNFR2 are expressed by the intraocular pigment epithelial cells. Further, these cells can produce TNF-α and the matrix metalloprotease, which can cleave TNF-α from the transmembrane form into a soluble form circulating within the eye. These factors constitute the basis through which TNF-α can cause intraocular inflammation. TNF-α is of vital importance for the intraocular immune reaction, which is referred to as “anterior chamber associated immune deviation” and for the autoregulation of intraocular cell apoptosis (MacEwan, 2002).

Although uveitis represents a group of intraocular inflammatory conditions, each with its own phenotypic heterogeneity, the commonality is the increased expression of TNF-α in both the serum and aqueous humor. Over the past decade, studies have increasingly emphasized the effectiveness of anti-TNF-α agents for patients with uveitis. Although there are numerous studies on TNF-α in various autoimmune diseases, the pathological mechanism and research progress with respect to the role of TNF-α in uveitis have not been reviewed. Thus, in this review, we aimed to provide a background of the role of TNF-α in the immunopathogenesis of uveitis and an account of the progress from bench to clinical research progress to better guide TNF-α-based therapeutics for uveitis.

## The Origin and Biology of Tumor Necrosis Factor-Alpha

TNF-α is a cytokine with diverse functions, including inflammation, immunity, cellular communication, cell differentiation, cell death, and survival, and a variety of signaling pathways. Although TNF was identified as early as 1975, the true identity of TNF was unclear until 1984 when Aggarwal et al. reported the isolation of cytotoxic factors, one of which was derived from macrophages, named TNF (Gray et al., 1984; Pennica et al., 1984; Kelker et al., 1985; O'Malley et al., 1988; Aggarwal et al., 2012). Using the same assays, Aggarwal et al. reported the isolation of a cytotoxic factor and named human TNF-α (Aggarwal et al., 1985). In 1990, two immunological TNF-binding proteins, namely 55 kDa (TNFR1) and 75 kDa (TNFR2), were identified, and subsequently, the cDNAs for both human proteins have been cloned (Hohmann et al., 1989; Schall et al., 1990).

TNF-α primarily exists as a trimeric transmembrane protein, transmembrane TNF-α (tmTNF-α), which is subsequently cleaved by TNF-α converting enzyme (TACE; also known as ADAM17) into a soluble form (sTNF-α) (Black et al., 1997). TNF-α has multifunctional bioactivity achieved by binding and activating two different receptors (TNFR1 and TNFR2). TNFR1, which is activated by sTNF-α and tmTNF-α, is ubiquitously expressed. TNFR1 bears the death domain that allows TNFR1 to organize the molecule TNF receptor-associated death domain (TRADD), which is a vital component of the TNFR1 signaling complex. In contrast, TNFR2 expression is limited to certain cell types (e.g., immune cells and endothelial cells). TNFR2 lacks a death domain resulting in its inability to recruit TRADD, and instead, it enlists TNFR-associated factor 1 (TRAF1) and TRAF2. TNFR2 is speculated to be activated primarily by tmTNF-α (Grell et al., 1995; Krippner-Heidenreich et al., 2002; Bystrom et al., 2018). However, there is evidence that sTNF-α might induce biological effects by transferring onto TNFR1 when binding to TNFR2. TNFRs can also be cleaved by TACE to produce soluble forms (sTNFRs), which bind to sTNF-α to exert effects. Studies have shown that sTNFRs are significantly increased in the ocular fluids of patients with active uveitis (Sugita et al., 2007). For TNF-α, the disparate distributions and binding characteristics to receptors are the pathological foundations for the occurrence and development of intraocular inflammation, which could indicate why systematic autoimmune diseases and uveitis have different responses to anti-TNF-α agents.

## Signaling Pathways Activated by Tumor Necrosis Factor-Alpha

**FIGURE 2:**
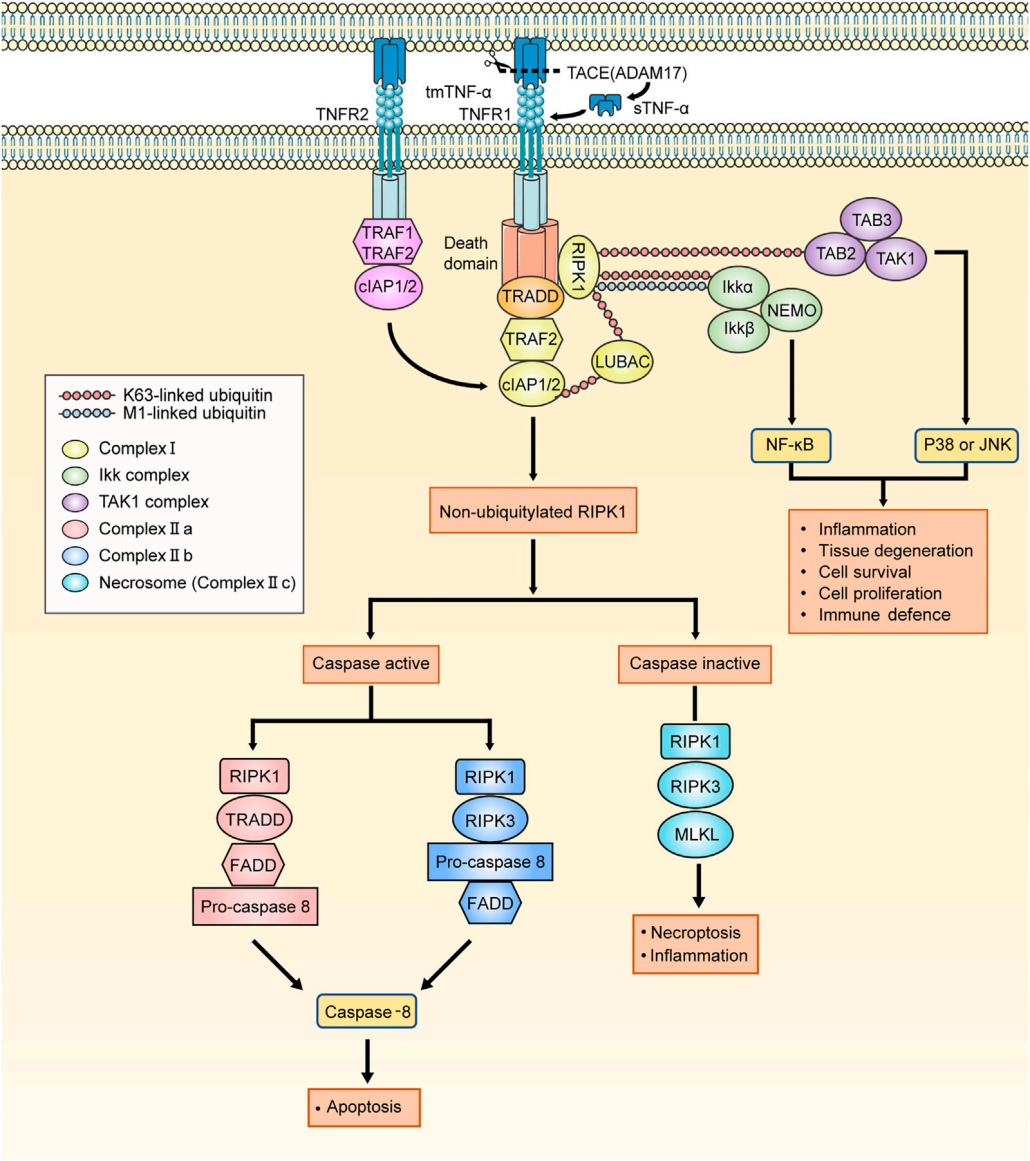
The signaling pathways activated by TNF-α. The tmTNF-α is cleaved by TACE into sTNF-α. The TNFR1 signaling is activated by both tmTNF-α and sTNF-α. When the binding of TNF-α to TNFR1, TNFR1 ligation leads to the recruitment of TRADD, RIPK1, LUBAC, TRAF2, cIAP1/2 and initiate the assembly of TNFR1 complex I. The TNFR2 signaling is almost activated by tmTNF-α. On account of the lack of TRADD, TNFR2 binds to TRAF1/2 directly to recruit cIAP1/2 and affiliate the TNFR1 signaling. The K63 ubiquitin ligase activities which is owned by cIAPs are required for LUBAC recruitment, and cIAPs add M1-linked linear polyubiquitin chains to RIPK1 which makes TAK1 complex and IKK complex assemble to respectively mediate JNK/p38 and NFκB pathways. And RIK1 deubiquitylates under conditions in which the K63-linked and M1-linked polyubiquitin chains are removed by the deubiquitylating enzyme CYLD from RIPK1. The residuum recruits TRADD, FADD and pro-caspase 8, thereby forming the complex IIa. When the cIAPs are depleted, there is no RIPK1 is deubiquitylated and leaves residuum to recruit FADD, pro-caspase 8 and RIPK3, assembling complex IIb. Following the assembly of complex II, pro-caspase 8 conducts autocatalytic cleavage, releasing active caspase 8 to trigger the implementation of the apoptotic program. When deubiquitylated RIPK1 exists but caspase is devitalized, RIPK1/3 cannot be inactivated. Instead, RIPK1 and RIPK3 cluster together to form the complex IIc (necrosome) and necroptosis program is initiated. TNFR1: TNF-α receptor 1; tmTNF-α: transmembrane TNF-α; sTNF-α: soluble TNF-α; TACE: the matrix metalloprotease TNF-α converting enzyme; TNF-α: tumour necrosis factor-alpha; TRADD: TNFR1-associated death domain protein; RIPK1: receptor-interacting serine/threonine-protein kinase 1; LUBAC: linear ubiquitin chain assembly complex; TRAF1/2: TNFR-associated factor 1/2; cIAP1/2: cellular inhibitor of apoptosis protein 1/2; TAK1 complex: TGF-β activated kinase 1 complex, consisting of TAK1, TAK1-binding protein 2 (TAB2) and TAB3; IKK complex: the complex comprising kinases IKKα and IKKβ, nuclear factor-κB (NF-κB) essential modulator (NEMO); JNK: Jun N-terminal kinase; CYLD: cylindromatosis; FADD: FAS-associated death domain protein.

When TNF-α binds to TNFR1, it assembles different signaling complexes consisting of the complexes I, IIa, IIb (ripoptosome), and IIc (necrosome), resulting in different functional outcomes (Pasparakis and Vandenabeele, 2015). TNF-α complex I signaling primarily mediates homeostatic bioactivities, which comprise tissue regeneration, cell proliferation and survival, inflammation, and immune defense. Similar effects can be caused by the combination of TNF-α and TNFR2, which may be related to the overlapping downstream pathways of TNFR1 signaling pathways. However, the formation of the complex IIa and IIb leads to the activation of a caspase cascade and results in apoptosis, whereas the necrosome induces necroptosis and inflammation. The signaling transduction pathways of the complexes are described briefly below and summarized in Figure 2.

### Tumor Necrosis Factor-Alpha Signaling in Complex I

With the binding of TNF-α to TNFR1, TRADD (Hsu et al., 1995), receptor-interacting protein kinase 1 (RIPK1), TRAF2, cellular inhibitor of apoptosis proteins 1 (cIAP1), cIAP2, and linear ubiquitin chain assembly complex (LUBAC) sequentially integrate into TNFR1 to form complex I (Silke and Brink, 2010; Brenner et al., 2015). The cIAPs have ubiquitin ligase activity, which is required for LUBAC recruitment, adding M1-linked linear polyubiquitin chains to RIPK1 (Komander and Rape, 2012). K63-polyubiquitylated RIPK1 associates with the TAK1 complex to activate Jun N-terminal kinase (JNK) and p38-mediated signaling. Furthermore, recruitment of the K63-polyubiquitylated RIPK1 and the IKK complex, which comprises kinases IKKα and IKKβ and nuclear factor-κB (NF-κB) essential modulator (NEMO), activates NF-κB-mediated anti-apoptotic signaling (Micheau and Tschopp, 2003). TNFR2 lacks the death domain sequence, rendering it incapable of recruiting TRADD, and instead, it recruits TRAF1/2 and cIAP1/2 directly. The TNFR2 signaling pathway will overlap with the subsequent TNFR1 signaling pathways here.

A recent study showed that MST1 negatively regulates TNF-α-induced NF-κB signaling by modulating LUBAC activity (Lee et al., 2019). Another report showed that TBK1 and IKKε (NEMO, as mentioned previously herein) prevent TNF-induced cell death via RIPK1 phosphorylation (Lafont et al., 2018). A similar study has shown that H-RN inhibits ocular inflammation in experimental autoimmune uveitis (EAU) by contributing to the attenuation of IKK complex activation and IκB degradation and significantly restraining the phosphorylation of NF-κB (Wang et al., 2014). More studies have been conducted to improve experimental uveitis by inhibiting the NF-κB signaling pathway, such as with lutein (Izumi-Nagai et al., 2007; Kijlstra et al., 2012), growth hormone (Liang et al., 2020), aminooxy-acetic acid (Meka et al., 2015; Mei et al., 2020), dehydroxymethylepoxyquinomicin (Ando et al., 2020), astaxanthin (Suzuki et al., 2006), silibinin (Chen et al., 2017), and aryl hydrocarbon receptor (Huang et al., 2018). Interestingly, a recent study showed that interleukin (IL)-17A inhibits the pathogenicity of Th17 cells by inducing the activation of IL-24 and the transcription factor NF-κB in EAU (Chong et al., 2020). However, clinical trials targeting IL-17A in uveitis have not been successful, which might be because the IL-17A-targeted drug improved EAU by inducing IL-24 *in vivo*, but silencing IL-24 in Th17 cells enhanced the disease. Some studies on inhibiting EAU by blocking the p38 signaling pathway, cannabidiol (El-Remessy et al., 2008), and IL-27 (Meka et al., 2015) have reported related results. These experiments have verified that the TNF-α signaling pathway is related to the pathogenesis of experimental uveitis, especially the NF-κB pathways (Li S. et al., 2010). The regulation of various signaling components in the TNF-α signaling pathways also seems to be promising for controlling the progression of uveitis when there is a poor response to TNF-α-agents. However, these ideas need to be verified with additional *in vivo* and *in vitro* experiments.

### Pathways Leading to Apoptosis and Necroptosis

RIPK1, as a pivotal molecular switch, determines whether TNF-α signaling pathways result in cell apoptosis or necroptosis (Ea et al., 2006). RIPK1 is not ubiquitinated under the action of the deubiquitination enzyme cylindromatosis (CYLD) (Kovalenko et al., 2003; Komander et al., 2009) or the depletion of cIAPs (Bertrand et al., 2008), and it recruits different signaling molecules to form complex IIa and IIb, respectively. Following the assembly of complex II, pro-caspase 8 conducts autocatalytic cleavage, releasing active caspase 8 to trigger the implementation of the apoptotic program (Wang et al., 2008). When deubiquitylated RIPK1 exists but caspase is deactivated, RIPK1 and RIPK3 cannot be inactivated. Instead, they cluster together to form complex IIc (necrosome) and the necroptosis program is initiated (He et al., 2009; Li et al., 2012). The level of RIPK3 in cells is responsible for cell necroptosis rather than apoptosis (Vandenabeele et al., 2010). TNF-α-induced cell necroptosis at various barrier surfaces impairs barrier function and leads to inflammation, such as retinal pigment epithelial (RPE) cells (Yumnamcha et al., 2019). However, it has been suggested that apoptosis and subsequent phagocytosis are of vital significance for the clearance of infiltrating cells from the eyes and the dissipation of EAU (Jha et al., 2007).

## Key Role of Tumor Necrosis Factor-Alpha in Understanding Uveitis

In patients with active uveitis or uveitis animal models, TNF-α levels in serum and aqueous humor are elevated, which is correlated with disease status (Fleisher et al., 1991; Kaufmann et al., 2012). TNF-α results in uveitis after intravitreal injection into the rabbit eye (El-Asrar et al., 2011). Evidence suggests a marked association between TNF-α and uveitis. TNF-α induces the release of secondary cytokines, such as IL-6 (Sironi et al., 1989; Sugita et al., 2007) and IL-8 (Sancéau et al., 1990), as well as a monocyte chemotactic and activating factor (Larsen et al., 1989a), to initiate a cascade of events integral to the inflammatory process. TNF-α also induces the release of bioactive lipids, such as eicosanoids (Larsen et al., 1989b; Zavoico et al., 1989), and platelet-activating factor (Bussolino et al., 1986; Camussi et al., 1987; Meyer et al., 1990) and increases the expression of adhesion molecules on vascular endothelial cells (e.g., vascular cell adhesion molecule-1,VCAM-1) (Pober et al., 1986; Bevilacqua et al., 1987; Valone and Epstein, 1988; Carlos et al., 1990). Some investigators reported that TNF-α plays an important role in the upregulation of matrix metalloproteinases (MMPs) in RPE cells and accounts for a directional shift in the balance between MMPs and tissue inhibitors of MMPs (Iademarco et al., 1995; Eichler et al., 2002). Moreover, MMPs, as a type of enzyme that degrades the extracellular matrix, are closely related to the integrity of the blood-retinal barrier (BRB) in uveitis patients (Li H. et al., 2010). T cells are important producers of TNF-α, and TNF-α regulates T cell responses (Nussenblatt, 1991; Cope et al., 1997). Studies have shown that anti-TNF-α therapy suppresses the differentiation of T-helper type 17 cells (Th17) and prevents severe eye inflammation (Masters et al., 2009). In brief, TNF-α, as a key link to intraocular inflammation, recruits leukocytes by mediating the production of intraocular chemokines, increases the adhesion of leukocytes to the vascular endothelium, enhances the antigen extraction ability of dendritic cells, activates macrophages and T cells, and eventually leads to the destruction of the BRB. The following part will summarize the previous research progress on the role of TNF-α in EAU in chronological order based on basic experiments, with emphasis on the aforementioned points.

### Progress on Tumor Necrosis Factor-Alpha in EAU

EAU was first described in 1965 (Wacker and Lipton, 1965; Sugita et al., 2012). It can be induced by many autoantigens of intraocular cells. Animal models have identified retinal S-antigen/arrestin (S-Ag) (Caspi, 2011), interphotoreceptor retinoid-binding protein (IRBP) (Bieganowska et al., 1997), rhodopsin (de Smet et al., 1990), opsin (Yamamoto et al., 1993), phosducin (Gery et al., 1994), recoverin (Dua et al., 1992), Rpe65 (Nityanand et al., 1993), melanin (Nakamura et al., 2005), and lens proteins and cellular retinaldehyde-binding protein (Broekhuyse et al., 1993) as “uveitogenic”. Now, EAU is generally used as experimental models of uveitis to study the immunopathologic mechanisms of human intraocular inflammatory diseases. Many studies have observed a constant increase in TNF-α expression in inflammatory cell infiltrates, not only in various models of experimental uveitis, but also in RPE and Müller cells, which causes these cells to possess uveitogenic properties and might decisively influence the course of EAU (de Kozak et al., 1997; de Smet and Chan, 2001; Holtkamp et al., 2001).

#### Tumor Necrosis Factor-Alpha and TNF-α Blockade in Different Animal Experimental Models

In 1993, a team observed that TNF-α could protect against the inflammatory processes of endotoxin-induced uveitis (EIU) (Huang et al., 2018). By contrast, Nakamura et al. reported that the injection of recombinant huTNF in mouse models increases susceptibility to EAU (Kasner et al., 1993). One experiment demonstrated that mice deficient in TNFR retain their susceptibility to EIU (Nakamura et al., 1994). However, another study indicated that mice with TNF receptor deficiency show decreased inflammation in an immune complex model of uveitis (Smith et al., 1998). In 1997, a study confirmed that TNF-α is not essential for inducing experimental autoimmune diseases (Brito et al., 1999), and a chronic low level of TNF-α might exert protective effects.

In 1996, a study showed that the neutralization of systemic TNF-α ameliorates the pathology of EAU, and interference with afferent processes, especially antigen priming, is important to protect against EAU through anti-TNF-α treatment (Frei et al., 1997). A similar result was observed in a 2001 study, in which IRBP-induced EAU in mice with a TNFR1-Ig fusion protein reduces damage to the retina (Sartani et al., 1996). However, TNF-α neutralization is ultimately not curative in experimental models of relapsing disease (Hankey et al., 2001). In 2003, Baker et al. (1994) identified that etanercept (an anti-TNF-α agent) decreases leukocyte rolling, leukocyte adhesion, and vascular leakage in a rat model of EIU. This outcome suggested that TNF-α is involved in the pathogenesis of uveitis and its potential use as a therapeutic drug to reduce ocular inflammation. In 2019, a study showed that intravitreal infliximab injection exacerbates inflammation in EIU models, whereas systemic infliximab treatment suppresses inflammation effectively and rapidly (Koizumi et al., 2003). It can be seen that the results of both TNF-α and TNF-α blocking experiments are inconsistent in different animal models. These opposite conclusions might be dependent on the experimental model, EAU or EIU. Moreover, these contradictory findings could suggest the different responses of patients with uveitis to certain therapies because of the diversity of uveitis pathogenesis.

#### Adhesion Molecule Regulation and BRB Rupture

In 1990, some investigators showed that TNF-α antagonists prevent adhesion molecule upregulation on the vascular endothelial cells in rheumatoid arthritis (RA) and experimental allergic encephalomyelitis (Ruddle et al., 1990; Liversidge et al., 2000). In 2011, investigators found that TNF-α expression decreases in aldehyde reductase-deficient mice, downregulating VCAM-1 expression (Elliott et al., 1994). In 2014, a study demonstrated that H-RN, a novel antiangiogenic peptide derived from hepatocyte growth factor which is an important angiogenic factor in vascular retinopathies, suppresses TNF-α-induced adhesion molecule expression (such as VCAM-1) in EAU (Wang et al., 2014). Further, silibinin was shown to prevent EIU and the subsequent production of ICAM-1 by blocking the NF-κB-dependent signaling pathway in 2017 (Chen et al., 2017).

In 1997, a study showed that TNF-α causes BRB rupture by opening tight junctions between retinal vascular endothelial cells and possibly by increasing transdermal vesicle transport in EAU (Yadav et al., 2011). In 2010, a team reported that TNF-α downregulates AQP1 protein expression in the retina, resulting in BRB breakdown (Luna et al., 1997). In 2017, chrysin (5,7-dihydroxyflavone) was reported to maintain the integrity of the BRB via suppression of the expression of inducible nitric oxide synthase (NOS) and macrophage infiltration in the retina, significantly decreasing the percentage of Th17 cells and CD4^+^ cells, increasing the percentage of Treg cells, and suppressing ocular inflammation during EAU (Motulsky et al., 2010). In 2018, a report indicated that aryl hydrocarbon receptor-knockout mice show a decrease in pro-inflammatory cytokines, such as TNF-α, thereby inhibiting retinal cell apoptosis and reducing BRB decomposition during EAU (Meng et al., 2017).

#### Effects of Tumor Necrosis Factor-Alpha on Macrophage and Th17 Activity

In 1998, Dick et al. observed that the inhibition of TNF-α activity protects against organ destruction without suppressing retinal T cell infiltration during EAU in Lewis rats. To demonstrate whether neutralizing TNF activity leads to a change in macrophage activation, some trials have used TNFR1, resulting in reduced nitrite in macrophages infiltrating the retina of the treated animal, thereby reducing target tissue damage and destruction (Dick et al., 1998). In these experiments, NOS2 inhibition induced by a nonspecific inhibitor of NOS resulted in a reduction in EAU (Robertson et al., 2003). The role of TNF-α in macrophages was also demonstrated in a 2009 study, which reported that high mobility group box 1 protein can stimulate TNF-α production in macrophages to promote and amplify ocular inflammation in EAU (Liversidge et al., 2002).

In 2007, Amadi et al. first described Th17 cells in EAU. They confirmed that IL-17 is increased in EAU, regulating TNF-α in retinal cells, suggesting a mechanism in which Th17 might contribute to ocular immunopathology (Watanabe et al., 2009). In 2019, a team reported that although TIPE2-deficient (TIPE2, one member of TNF-α-induced protein) T cells produce more IL-17, they do not migrate to the skin as efficiently. Instead, they migrate to the inflamed eye in a similar manner to TIPE2-deficient T cells and thus exacerbate the development of EAU in TIPE2-deficient mice but reduce the severity of psoriasis in these animals (Amadi-Obi et al., 2007).

## Tumor Necrosis Factor-Alpha as a Therapeutic Target for Uveitis

Systemic immunomodulatory therapy (IMT) has been used to treat specific patients with uveitis over the last decades. Corticosteroids are an important component of IMT and are also the first-line treatment for uveitis. However, patients with uveitis are at risk of long-term complications caused by long-term uncontrolled inflammation and corticosteroid therapy, which can reduce the treatment success rate for the disease itself. Therapeutic strategies have evolved over the last few years, and anti-TNF-α agents have become well accepted for the treatment of refractory uveitis. Anti-TNF-α agents have fewer adverse effects than corticosteroids. Studies have shown that when used properly, dependence on corticosteroids can be significantly reduced to prevent uveitis recurrence (Liu et al., 2019).

### Development of anti-TNF-α Agents in Uveitis

The first use of anti-TNF-α agents was reported in the 1980s in experimental models of sepsis (Beutler et al., 1985; Tracey et al., 1987; Calandra et al., 1991; Hu et al., 2020). In 1985, Feldmann et al. identified TNF-α as a therapeutic target for RA and reported the first proof of concept trials (Feldmann and Maini, 2003). In 1991, Keffer et al. reported the effectiveness of anti-TNF-α therapy for arthritis (Keffer et al., 1991). The success of phase I/II trials of anti-TNF-α antibodies announced in 1992 contributed to the performance of clinical trials for other chronic diseases. Since the first reported use of infliximab in 2001 for uveitis treatment, several new anti-TNF-α agents have been developed for the treatment of refractory uveitis (Muñoz-Fernández et al., 2001; Sfikakis et al., 2001). Four monoclonal anti-TNF-α antibodies, namely, infliximab (IFX; Remicade®), adalimumab (ADA; Humira®), golimumab (GOL; Simponi®), and certolizumab pegol (CZP; Cimzia®), are available. Etanercept (Enbrel®) is the only commercially available receptor fusion protein (Sandborn et al., 2001). In 2011, Cordero-Coma et al. first reported two cases of treatment with GOL, which both achieved satisfactory results (Tracey et al., 2008; Cordero-Coma et al., 2011). In 2016, the United States Food and Drug Administration (FDA) approved ADA as the first anti-TNF-α agent for the treatment of non-infectious intermediate, posterior, and panuveitis (Hasegawa et al., 2019). In the same year, clinical trials were performed on the effectiveness of CZP for refractory spondyloarthritis-related uveitis, but no significant advantages were found over other anti-TNF-α agents (Rudwaleit et al., 2016). Different inhibitors have different functional profiles. IFX, ADA, and GOL are humanized monoclonal antibodies, whereas CZP is a monovalent fragment linked to polyethylene glycol. ADA and GOL are fully human monoclonal antibodies; however, IFX is a chimeric protein with both human and murine components. The lack of the fragment crystallizable (Fc) portion suppresses the high immunogenicity of CZP and makes it less likely to cross the placenta in pregnant patients. Etanercept is a recombinant fusion protein composed of the extracellular portions of TNFR2 combined with the Fc portion of human immunoglobulin G-1. The most frequent side effect was determined to be infusion reaction, with infectious diseases including tuberculosis being second most common; the occurrence of demyelinating or autoimmune diseases was seldom reported. The associated risk of cancer has been debated. To date, anti-TNF-α agents have made more progress for uveitis treatment. The different characteristics of anti-TNF-α agents derived from clinical trials are summarized as below (summarized in Supplementary Table S1).

#### ADA (Humira®)

The advantages of ADA are listed as follows:

1) Compared with IFX, ADA is a fully human monoclonal antibody that causes almost no allergic reactions, and subcutaneous injection is safer and more convenient than intravenous injection (Ming et al., 2018).
2) During steroid tapering, ADA significantly reduces the relapse rate, visual deterioration, and anterior chamber flare, and has relatively good tolerance (Díaz-Llopis et al., 2012; Jaffe et al., 2016).
3) The use of ADA in the treatment of uveitis associated with Behçet’s disease (BD) is not affected by the concomitant application of antirheumatic agents (Nguyen et al., 2016; Fabiani et al., 2018).
4) Numerous studies have shown that ADA is superior to immunosuppressive agents in decreasing the relapse rate and occurrence of retinal vasculitis and improving visual acuity (Sota et al., 2021).
5) ADA is safe and efficacious for the treatment of non-infectious uveitis in elderly patients (Moll-Udina et al., 2020).
6) ADA seems to be associated with better outcomes after follow-up, although both IFX and ADA are efficacious for refractory BD-related uveitis (Atienza-Mateo et al., 2019).
7) ADA plus conventional therapy outperforms conventional therapy alone in patients with retinal vasculitis due to refractory BD-related uveitis (Yang et al., 2021a; Yang et al., 2021b).
8) In children and adolescents with active juvenile idiopathic arthritis (JIA)-related uveitis, the treatment failure rate of ADA is lower than that of the placebo (Ramanan et al., 2017; Angeles-Han et al., 2019).

The disadvantages of ADA are as follows:

1) Adverse events were reported in patients who received ADA (Díaz-Llopis et al., 2012). The most frequently reported treatment-emergent adverse event is infection (Al-Janabi et al., 2020).
2) The use of ADA for undifferentiated uveitis might result in premature discontinuation on account of side effects (Al-Janabi et al., 2020; Llorenç et al., 2020).

The indications are as follows:

1) Non-infectious uveitis, intermediate uveitis, posterior uveitis, and panuveitis in adult patients with underreaction and contraindications to steroids, as well as steroid dependence in Europe (Leclercq et al., 2020).
2) Non-infectious uveitis, intermediate uveitis, posterior uveitis, and panuveitis in adult patients in the United States (Leclercq et al., 2020).
3) As a first-line immunomodulator for the treatment of ophthalmic manifestations of BD (Touhami et al., 2019).
4) As a second-line immunomodulator for the treatment of uveitis associated with JIA (Angeles-Han et al., 2019; Llorenç et al., 2020).
5) ADA is approved for RA, ulcerative colitis, psoriatic arthritis, ankylosing spondylitis (AS), Crohn’s disease, and plaque psoriasis in adults (Llorenç et al., 2020).

#### IFX (Remicade®)

The advantages of IFX are listed as follows:

1) IFX showed commendable efficacy for refractory non-infectious uveitis and severe uveitis cases associated with BD whether it was used as monotherapy or with other immunosuppressive agents (Vallet et al., 2015; Vallet et al., 2016).
2) IFX showed a significantly higher capacity to resolve macular edema in treating sight-threatening retinal vasculitis when compared with the effects of ADA (Levy-Clarke et al., 2014).
3) A report indicated that IFX is effective as a treatment for visually threatening refractory posterior uveitis (Joseph et al., 2003).
4) Multiple studies have shown that IFX is superior to immunosuppressive agents in reducing recurrence rates and ameliorating visual acuity (Vallet et al., 2015).
5) Arida et al. reported that 40% of BD cases remained in remission 3 years after the discontinuation of IFX (Vallet et al., 2015). In the event of relapse, good response rates were obtained after the resumption of IFX therapy (Markomichelakis et al., 2011).

The disadvantages of the IFX are listed as follows:

1) Tolerance is low owing to the relatively frequent infusion reactions (Lichtenstein et al., 2015; Leclercq et al., 2020).
2) Tuberculosis as an adverse effect was reported in patients treated with IFX (Tugal-Tutkun et al., 2005).
3) One study reported a higher rate of IFX toxicity in patients with uveitis (Markomichelakis et al., 2011).

The indications are as follows:

1) Numerous experts have recommended IFX as first-line therapy for visually threatening BD (macular ischemia, cystoid macular edema, serious vasculitis, monophthalmic patients) (Arida et al., 2011; Hatemi et al., 2018).
2) As a second-line immunomodulator for the treatment of uveitis related to JIA (Angeles-Han et al., 2019).
3) For the treatment of severe ocular inflammatory conditions including posterior uveitis, panuveitis, severe uveitis associated with seronegative spondyloarthropathy, and scleritis in patients requiring immunomodulation (Levy-Clarke et al., 2014).
4) Infliximab is authorized by the FDA for the treatment of RA, AS, Crohn’s disease, psoriatic arthritis, plaque psoriasis in adults, and ulcerative colitis (Sobrin et al., 2007; Arida et al., 2011).

#### GOL (Simponi®)

The advantages of the GOL are listed as follows:

1) Compared with IFX, GOL is a fully human monoclonal antibody that causes almost no allergic reactions (Ming et al., 2018).
2) GOL is effective in improving visual acuity and controlling ocular inflammation (Cordero-Coma et al., 2014).
3) GOL has been proven to be conducive to AS-related anterior uveitis, ameliorating macular edema and inflammation, and decreasing the relapse rate (Calvo-Río et al., 2016; Fabiani et al., 2016).
4) The control of intraocular inflammation with multi-refractory uveitis associated with BD (Hatemi et al., 2018).
5) GOL represents an efficacious and secure therapy choice for uveitis with a significant reduction in the frequency of ocular flares while preserving visual function with a satisfactory long-term retention rate (Fabiani et al., 2019).
6) The effective treatment of JIA and idiopathic retinal vasculitis by GOL has been reported, whereas other anti-TNF-α agents are ineffective (Tosi et al., 2019).

#### CZP (Cimzia®)

The advantages of the CZP are listed as follows:

1) CZP can be an effective alternative to long-lasting chronic relapsing uveitis (Llorenç et al., 2016).
2) Some studies have shown a significant decrease in ocular flares with a satisfactory long-term retention rate with CZP compared to that with placebo (Tosi et al., 2019).
3) A national multicenter observational study supported the efficacy of CZP for the management of uveitis during pregnancy (Prieto-Peña et al., 2021). In terms of pregnancy safety, CZP displayed advantageous properties over other anti-TNF-α agents because of its limited transport across the placenta (Mariette et al., 2018).
4) One study showed that the relative *in vitro* neutralizing potency is higher for CZP than for ADA (Berkhout et al., 2020).
5) One study observed positive outcomes using CZP as therapy for patients with refractory, non-infectious uveitis when other anti-TNF-α agents proved inadequate or when tolerance issues were present (Sharon and Chu, 2020).

#### Etanercept (Enbrel®)

The disadvantages of etanercept are listed as follows:

1) Owing to its poor intraocular permeability and limited effectiveness, it is not recommended for uveitis (Dick et al., 2018).
2) Granulomatosis, as a side effect, has been reported in the treatment of uveitis with etanercept (Leal et al., 2019).
3) Meta-analyses have shown that etanercept is inferior to other anti-TNF-α agents for uveitis treatment (Leal et al., 2019).
4) Paradoxical occurrences of uveitis have also been reported after etanercept administration in patients with AS-related acute anterior uveitis (Fabiani et al., 2016).
5) Etanercept might be less efficient than other anti-TNF-α agents in decreasing the risk of HLA-B27-related acute anterior uveitis in patients with spondyloarthritis (Mitulescu et al., 2018).

The indications are as follows:

1) Etanercept received FDA approval for RA, polyarticular JIA, AS, psoriatic arthritis, and plaque psoriasis (in patients aged 17 years and older) (Fabiani et al., 2016).
2) International guidelines concluded that the use of etanercept for the treatment of uveitis is not supported (Dick et al., 2018).

## Conclusion

Inefficiently controlled or untreated uveitis is one of the primary causes of blindness in developed countries. Corticosteroids remain the first-line treatment; however, their chronic use can result in side effects. These complications have led investigators to seek corticosteroid-sparing treatments. Although uveitis represents a group of intraocular inflammatory conditions with distinct phenotypic heterogeneity, its common feature is increased expression of TNF-α in both the serum and aqueous humor. Over the past decade, studies have increasingly emphasized the effectiveness of anti-TNF-α agents for patients with uveitis. However, the lack of clinical trials and the rarity and heterogeneity of uveitis make their utilization in ophthalmology more challenging, particularly for first-line therapy.

Most international studies have focused on ADA and IFX, which are the most commonly recently employed biological agents for patients with uveitis. Authoritative experts recommended the use of ADA in cases of nullity or intolerance to immunosuppressive agents for non-infectious non-anterior uveitis. IFX was proposed as a first-line treatment for sight-threatening uveitis associated with BD. Nevertheless, knowing which of the two has a better effect in combating uveitis is an unmet demand. ADA is well tolerated with acceptable side effect profiles, and its costs have also decreased to acceptable levels. These properties make it an excellent option as second-line and reserved steroid therapy for uveitis. However, whether the earlier introduction of ADA would confer additional benefits in the management of uveitis and the preservation of visual function is unclear. GOL seems to have more evident advantages as a therapy for spondylitis-related uveitis. In terms of pregnancy safety, CZP has favorable characteristics over other anti-TNF-α agents owing to its limited transport across the placenta.

Furthermore, there are still many questions regarding the use of anti-TNF-α agents as a therapy for uveitis, including the following: treatment duration, when to stop using, the necessity to monitor drug levels regularly, alternative biological agents if anti-TNF-α failure occurs, how to reduce the immunogenicity against anti-TNF-α molecules, and how to ameliorate efficacy. Moreover, treatment failure when using one anti-TNF-α agent does not indicate that other agents in the same group will also be ineffective. Some studies have reported that agents in a group can be replaced with each other by changing novel routes of drug administration to less intense places such as subcutaneous injections. The development of monoclonal antibodies that simultaneously recognize multiple targets allows for more effective treatment of uveitis at a lower dose than that with any single biological drug. Alternatively, a secure and efficient sustained-release device can be developed that will enable the topical treatment of idiopathic immune-mediated uveitis with immunomodulators, including biological response modifiers. The pathogenic effect of TNF-α is caused by complex signaling pathways composed of cascades of signaling molecules. When the efficacy of anti-TNF-α agents is not good, changing therapeutic targets to different signaling molecules in the pathway is also a good alternative. However, these ideas need to be verified with additional *in vivo* and *in vitro* experiments.
