# Corrigendum: TNF-α in Uveitis: From Bench to Clinic

**DOI:** 10.3389/fphar.2022.817235

**Published:** 2022-02-25

**Authors:** Qi Jiang, Zhaohuai Li, Tianyu Tao, Runping Duan, Xianggui Wang, Wenru Su

**Affiliations:** ^1^ State Key Laboratory of Ophthalmology, Zhongshan Ophthalmic Center, Sun Yat-sen University, Guangzhou, China; ^2^ Eye Center of Xiangya Hospital, Central South University, Changsha, China; ^3^ Hunan Key Laboratory of Ophthalmology, Changsha, China

**Keywords:** uveitis, TNF-α, experimental autoimmune uveitis (EAU), anti-TNF-α agents, infliximab, adalimumab, golimumab, certolizumab pegol

There is an error in the **Funding** statement. The correct **Funding** statement appears below.

The authors apologize for this error and state that this does not change the scientific conclusions of the article in any way. The original article has been updated.

