# Supplementary material for: TNF-α in Uveitis: From Bench to Clinic
Source: Front Pharmacol. 2021 Nov 2;12:740057. doi: 10.3389/fphar.2021.740057 (PMC8592912; doi:10.3389/fphar.2021.740057)
Supplement: Supplementary file 1 [file Table1.DOCX]

Supplementary Material

# Supplementary Table 1.

Table 1. The different characteristics of anti-TNF-α agents derived from clinical trials.

| **ADA(Humira®)** | |
| --- | --- |
| Advantages | No allergic reactions, and subcutaneous injection is safer and more convenient (Ming et al., 2018). |
|  | It Significantly reduces the relapse rate, visual deterioration, and anterior chamber flare, and has relatively good tolerance during steroid tapering (Díaz-Llopis et al., 2012; Jaffe et al., 2016). |
|  | The treatment of uveitis associated with BD is not affected by the concomitant application of antirheumatic agents (Nguyen et al., 2016; Fabiani et al., 2018). |
|  | It is superior to immunosuppressive agents in decreasing the relapse rate and occurrence of retinal vasculitis and improving visual acuity (Sota et al., 2021). |
|  | It is safe and efficacious for the treatment of non-infectious uveitis in elderly patients (Moll-Udina et al., 2020). |
|  | Better outcomes after follow-up, although both IFX and ADA are efficacious for refractory BD-related uveitis (Atienza-Mateo et al., 2019). |
|  | ADA plus conventional therapy outperforms conventional therapy alone in patients with retinal vasculitis due to refractory BD-related uveitis (Yang et al., 2021a; Yang et al., 2021b). |
|  | The treatment failure rate is lower than that of the placebo in children and adolescents with active JIA-related uveitis (Ramanan et al., 2017; Angeles-Han et al., 2019). |
| Disadvantages | Adverse events were reported in patients who received ADA (Díaz-Llopis et al., 2012). The most frequently reported treatment-emergent adverse event is infection (Al-Janabi et al., 2020). |
|  | The use of ADA for undifferentiated uveitis might result in premature discontinuation on account of side effects (Al-Janabi et al., 2020; Llorenç et al., 2020). |
| Indications | Non-infectious uveitis, intermediate uveitis, posterior uveitis, and panuveitis in adult patients with underreaction and contraindications to steroids, as well as steroid dependence in Europe (Leclercq et al., 2020). |
|  | Non-infectious uveitis, intermediate uveitis, posterior uveitis, and pan uveitis in adult patients in the United States (Leclercq et al., 2020). |
|  | As a first-line immunomodulator for the treatment of ophthalmic manifestations of BD (Touhami et al., 2019). |
|  | As a second-line immunomodulator for the treatment of uveitis associated with JIA (Angeles-Han et al., 2019; Llorenç et al., 2020). |
|  | It is approved for RA, AS, ulcerative colitis, psoriatic arthritis, Crohn’s disease, and plaque psoriasis in adults (Llorenç et al., 2020). |
| **IFX (Remicade®)** | |
| Advantages | It showed the commendable efficacy for refractory non-infectious uveitis and severe uveitis cases associated with BD whether it was used as monotherapy or with other immunosuppressive agents (Vallet et al., 2015; Vallet et al., 2016). |
|  | It showed the significantly higher capacity to resolve macular edema in treating sight-threatening retinal vasculitis when compared with the effects of ADA (Levy-Clarke et al., 2014). |
|  | It is effective as a treatment for visually threatening refractory posterior uveitis (Joseph et al., 2003). |
|  | It is superior to immunosuppressive agents in reducing recurrence rates and ameliorating visual acuity (Vallet et al., 2015). |
|  | 40% of BD cases remained in remission 3 years after the discontinuation of IFX. In the event of relapse, good response rates were obtained after the resumption of IFX therapy (Markomichelakis et al., 2011; Vallet et al., 2015). |
| Disadvantages | Tolerance is low owing to the relatively frequent infusion reactions (Lichtenstein et al., 2015; Leclercq et al., 2020). |
|  | Tuberculosis as an adverse effect was reported (Tugal-Tutkun et al., 2005). |
|  | A higher rate of IFX toxicity was reported (Markomichelakis et al., 2011). |
| Indications | Numerous experts have recommended IFX as a first-line therapy for visually threatening BD (macular ischemia, cystoid macular edema, serious vasculitis, monophthalmic patients) (Arida et al., 2011; Hatemi et al., 2018). |
|  | As a second-line immunomodulator for the treatment of uveitis related to JIA (Angeles-Han et al., 2019). |
|  | For the treatment of severe ocular inflammatory conditions including posterior uveitis, pan uveitis, severe uveitis associated with seronegative spondyloarthropathy, and scleritis in patients requiring immunomodulation (Levy-Clarke et al., 2014). |
|  | It is authorized by the FDA for the treatment of RA, AS, Crohn’s disease, psoriatic arthritis, plaque psoriasis in adults, and ulcerative colitis (Sobrin et al., 2007; Arida et al., 2011). |
| **GOL (Simponi®)** | |
| Advantages | As a fully human monoclonal antibody causes, it almost no allergic reactions compared with IFX (Ming et al., 2018). |
|  | It is effective in improving visual acuity and controlling ocular inflammation (Cordero-Coma et al., 2014). |
|  | It is conducive to AS-related anterior uveitis, ameliorating macular edema and inflammation, and decreasing the relapse rate (Calvo-Río et al., 2016; Fabiani et al., 2016). |
|  | The control of intraocular inflammation with multi-refractory uveitis associated with BD (Hatemi et al., 2018). |
|  | It is an efficacious and secure therapy choice for uveitis with a significant reduction in the frequency of ocular flares while preserving visual function with a satisfactory long-term retention rate (Fabiani et al., 2019). |
|  | The effective treatment of JIA and idiopathic retinal vasculitis by GOL has been reported, whereas other anti-TNF-α agents are ineffective (Tosi et al., 2019). |
| **CZP (Cimzia®)** | |
| Advantages | An effective alternative to long-lasting chronic relapsing uveitis (Llorenç et al., 2016). |
|  | A significant decrease in ocular flares with a satisfactory long-term retention rate with CZP compared to that with placebo (Tosi et al., 2019). |
|  | A national multicenter observational study supported the efficacy of CZP for the management of uveitis during pregnancy (Prieto-Peña et al., 2021). In terms of pregnancy safety, CZP displayed advantageous properties over other anti-TNF-α agents because of its limited transport across the placenta (Mariette et al., 2018). |
|  | The relative in vitro neutralizing potency is higher for CZP than ADA (Berkhout et al., 2020). |
|  | A study observed positive outcomes using CZP as therapy for patients with refractory, non-infectious uveitis when other anti-TNF-α agents proved inadequate or when tolerance issues were present (Sharon and Chu, 2020). |
| **Etanercept (Enbrel®)** | |
| Disadvantages | Owing to its poor intraocular permeability and limited effectiveness, it is not recommended for uveitis (Dick et al., 2018). |
|  | Granulomatosis, as a side effect, has been reported in the treatment of uveitis with etanercept (Leal et al., 2019). |
|  | Meta-analyses have shown that etanercept is inferior to other anti-TNF-α agents for uveitis treatment (Leal et al., 2019). |
|  | Paradoxical occurrences of uveitis have also been reported after etanercept administration in patients with AS-related acute anterior uveitis (Fabiani et al., 2016). |
|  | It might be less efficient than other anti-TNF-α agents in decreasing the risk of HLA-B27-related acute anterior uveitis in patients with spondyloarthritis (Mitulescu et al., 2018). |
| Indications | Etanercept received FDA approval for RA, polyarticular JIA, AS, psoriatic arthritis, and plaque psoriasis (in patients aged 17 years and older) (Fabiani et al., 2016). |
|  | International guidelines concluded that the use of etanercept for the treatment of uveitis is not supported (Dick et al., 2018). |

ADA: adalimumab; BD: Behçet’s disease; IFX: infliximab; JIA: juvenile idiopathic arthritis; RA: rheumatoid arthritis; AS: ankylosing spondylitis; FDA: the United States Food and Drug Administration; GOL: golimumab; CZP: certolizumab pegol.
